# Biomolecular Interaction Prediction in the Pre‐ and Post‐AlphaFold Era: The 8th CAPRI Evaluation

**DOI:** 10.1002/prot.70018

**Published:** 2025-07-14

**Authors:** Marc F. Lensink, Nessim Raouraoua, Guillaume Brysbaert, Sameer Velankar, Shoshana J. Wodak, Alexandre M. J. J. Bonvin

**Affiliations:** ^1^ University of Lille, CNRS UMR8576 UGSF, Unite de Glycobiologie Structurale et Fonctionnelle Lille France; ^2^ European Molecular Biology Laboratory European Bioinformatics Institute (EMBL‐EBI), Wellcome Trust Genome Campus Cambridge UK; ^3^ VIB‐VUB‐Structural Biology Research Center Brussels Belgium; ^4^ Bijvoet Centre for Biomolecular Research, Faculty of Science – Chemistry Utrecht University Utrecht the Netherlands

**Keywords:** artificial intelligence, biomolecular interaction, blind prediction, CAPRI, protein complexes, protein docking, scoring, structure prediction

## Abstract

We report on the 8th CAPRI Evaluation period, capturing the assessment of CAPRI Rounds 47 to 55 (excluding the CASP and COVID‐related Rounds), which have witnessed the transition to AI‐driven prediction tools such as AlphaFold and related alternatives. The prediction Rounds in this evaluation are characterized by a high level of difficulty due to various factors, including the nature of the targets, the intricacy of the interfaces to be predicted, and conformational changes. A total of 11 targets encompassing 21 interfaces, mostly in the difficult prediction category, were evaluated. While a retrospective analysis reveals a strong performance of AlphaFold on those targets, human predictors still outperform AI on difficult targets, particularly those involving antibodies and nucleic acids. Almost 25 years after its birth, CAPRI remains a vibrant and collaborative initiative with active participation from approximately 50 predictor and scorer groups and 10 servers. Continued contributions from experimentalists providing targets to such blind experiments, and further advances in AI, sampling strategies, and improvement in scoring methods will be key to overcoming remaining structural prediction challenges in complex biomolecular systems.

## 1 Introduction

Biomolecular interactions govern a wide range of cellular and intercellular activities. Characterizing these interactions at the atomic level is essential for understanding the mechanisms underlying health and disease and for developing new therapeutics. Experimental structural biology, with X‐ray crystallography, Nuclear Magnetic Resonance (NMR) and cryo‐electron microscopy (cryo‐EM) and tomography (cryo‐ET) is continuously providing atomic insights into the three‐dimensional (3D) structure of those interactions [1]. Over the years, many other experimental methods have been providing pieces of this interaction puzzle. Similarly, bioinformatics approaches have also evolved, from predicting binding sites to predicting intermolecular contacts from co‐evolutionary signals. These puzzle pieces on their own are not sufficient to solve the 3D structure of a complex and must therefore be combined with computational methods, which has given rise to the field of integrative structural biology [2, 3, 4].

On the computational side, docking methods that are generating 3D models of biomolecular complexes from their free components have contributed since ~50 years to populating the interaction landscape. In order to provide an unbiased assessment of the field, CAPRI (Critical Assessment of PRedicted Interactions) was established in 2001 as a community‐wide initiative, inspired by CASP (Critical Assessment of protein Structure Prediction) [5]. Its main goal is to assess the current state of the art and catalyze progress in computational methodology for modeling protein complexes. It has done so by offering participants the opportunity of testing their algorithms in blind predictions of experimentally determined 3D structures of protein complexes, the ‘targets', provided to CAPRI prior to publication. CAPRI's focus over the years has been mainly on protein–protein assemblies [6], but other types of complexes have been included, such as protein‐nucleic acids, protein‐peptides and protein‐glycans [7, 8, 9, 10, 11].

Due to the paucity of available protein complexes that can be used as targets, a CAPRI prediction Round is initiated each time a target (or a few targets) becomes available and completed three to 6 weeks later. Targets are structures of protein complexes/assemblies offered in strict confidence to CAPRI by structural biologists prior to publication. Registered participants are invited to predict the 3D structure of the target protein assembly starting typically from sequence information alone. A Round also includes a scoring challenge in which the correct assembly models must be identified out of a pool of decoys (incorrect models) submitted by the participating predictors. Groups can participate in either or both challenges (as predictors and scorers). Servers can also participate in CAPRI and do so with a much shorter prediction time of at most 3 days.

Historically, most predictions in CAPRI originate from docking and related conformational sampling approaches, often using shape complementarity with various other energy‐based terms to score the models [5, 12]. With the publication of AlphaFold2 (AF) in 2021 [13], artificial intelligence (AI) quickly entered the CAPRI prediction field. This catalyzed the development of many other AI‐based prediction methods like for example, RoseTTafold [14], AF‐multimer [15], OpenFold [16], and many others. The recent release of AlphaFold3 opened the route to the prediction of a variety of complexes including those with nucleic acids and small molecules [17], although with more restrictive access and licensing terms, and was quickly followed by the release of more permissive alternatives, such as Boltz‐1 and Chai‐1 [18, 19]. These AI methods have demonstrated excellent performance for protein–protein complex prediction in cases where coevolution can be used, but still often struggle with, for example, antibody and protein nucleic acids complexes. Some of these limitations can be lifted by massively increasing the sampling, which can be performed with tools like MassiveFold [20], but this comes at very high computational costs.

In the current manuscript, we describe CAPRI Rounds 47 to 55 (excluding the CASP Rounds 50 and 54, and the COVID effort—Round 51) which have, halfway, witnessed the rise of AI. As of Round 53, AI prediction methods have been integrated into most prediction pipelines of the participating groups. These Rounds were discussed at the 8th CAPRI Evaluation Meeting, which took place in Grenoble in February 2024. AlphaFold2 itself is now used by the CAPRI assessors to define a prediction accuracy baseline and assess the difficulty of a target. In the following, we first briefly present the targets and the assessment methodology and then discuss the overall results and groups' performance, distinguishing between the pre‐ and post‐AlphaFold era.

## 2 The Targets

Information on the targets of these Rounds is summarized in Table 1. The targets are listed in chronological order. With one exception, all targets were deemed Difficult. The modeling tasks were various: prediction of domain organization, homo‐ and hetero‐dimer interaction, loop modeling, and protein‐DNA, protein‐nanobody, and antibody–antigen interaction. The 10 targets are depicted in Figure 1.

**TABLE 1:** The CAPRI targets of the 8th Evaluation period.

Round	Target	Stoichiometry	#int, #tgt	Area/Å^2^	Experimental technique	Resolution	PDB	Type	Difficulty
Pre‐AlphaFold targets									
R47	T160	A	6, 6	150–500	X‐ray	2.7 Å	6HHU	Domain organization	Difficult
		A:nb	1, 1	950			6HUU	Nanobody binding	Difficult
		A:nb	1, 1	600			6HUU	Nanobody binding	Difficult
R48	T161	A2	1, 1	7000	X‐ray	2.64 Å	6Y2P	Toxin homodimer	Difficult
	T162	AB	1, 1	1750	X‐ray	2.64 Å	6Y2P	Enzyme/inhibitor	Difficult
R49	T163	A2B2	5, 3	500–1400	X‐ray	2.42 Å	6R17	Protein/protein	Difficult
R52	T186	ABCDEFG	3, 2	3750	cryo‐EM	2.1 Å	7NZ1	Loop modeling	Difficult
Post‐AlphaFold targets									
R53	T187	A2:DNA	1, 1	7900	cryo‐EM	3.1 Å	7QD5	Protein/DNA	Difficult
	T188	A2:DNA	1, 1	7900	cryo‐EM	3.1 Å	7QD5	Protein/DNA (bound)	Difficult
R55	T231	A:HL	1, 1	900	X‐ray	1.16 Å	8RMO	Antibody/peptide	Difficult
	T232	A:B	1, 1	1500	cryo‐EM	N/A	N/A	Protein/protein	Easy
	T233	AB:HL	1, 1	1650	X‐ray	N/A	N/A	Antibody/antigen	Difficult
	T234	AB:HL	1, 1	1350	X‐ray	N/A	N/A	Antibody/antigen	Difficult

*Note:* Targets are listed in chronological order. Rounds 50 and 54 were joint CASP‐CAPRI rounds whose results are detailed elsewhere [21, 22]. #int. stands for the number of interfaces, and #tgt for the number of (sub)‐targets. The difficulty level is assigned manually, based on template availability (pre‐AlphaFold) or AlphaFold performance (post‐AlphaFold).

**FIGURE 1:**
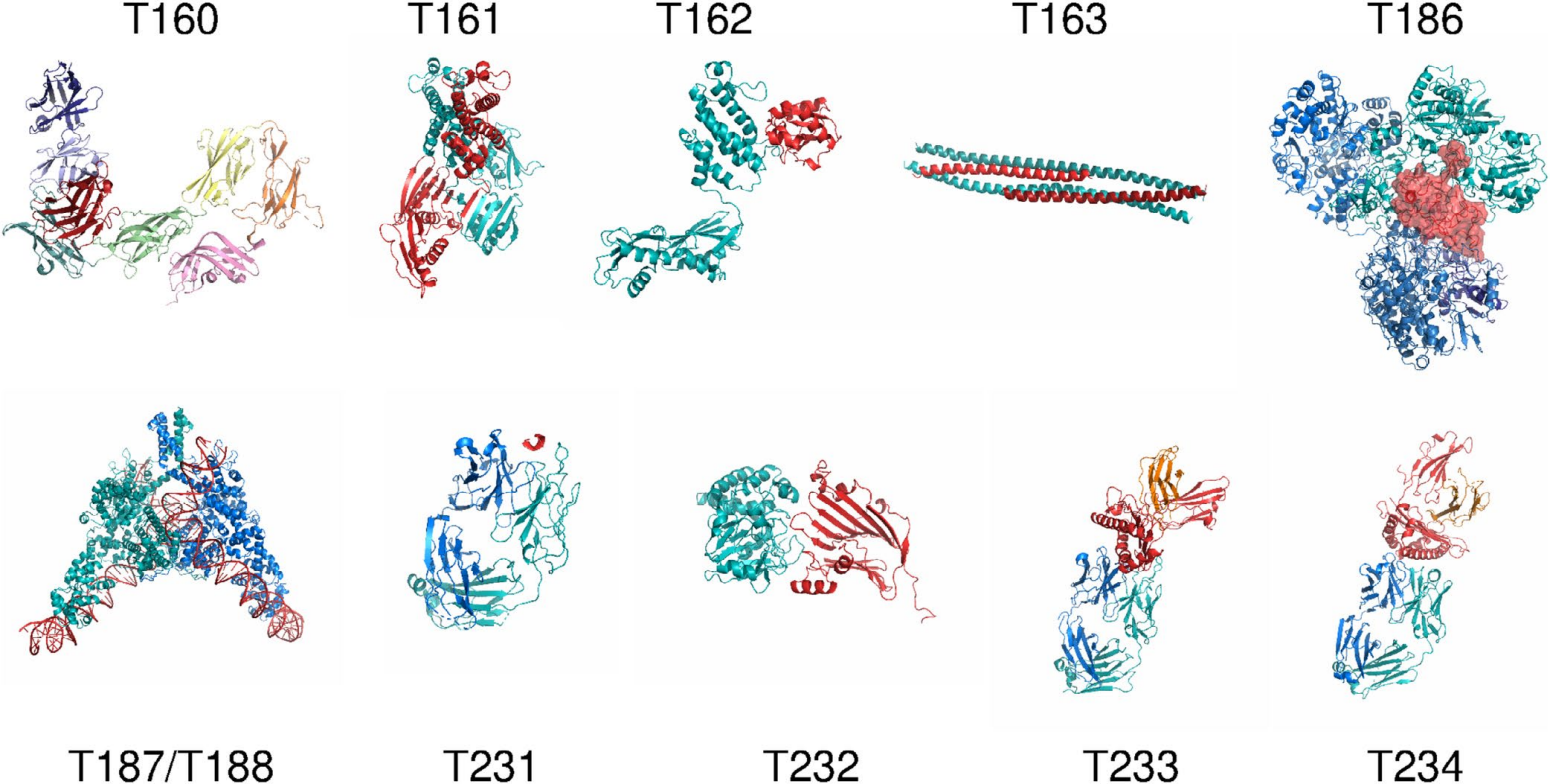
The 10 CAPRI targets of the 8th Evaluation period. The 10 targets (see also Table 1) are labeled by their CAPRI target number. For T160 the individual domains D1–D6 are colored lightblue‐cyan‐green‐yellow‐orange‐pink and the two nanobodies darkblue and red. For T186 the accessible surface area of the loop to be modeled is shown for visual clarity. For all targets (except T160) the larger entity is colored blue/cyan and the smaller entity red/orange. Images were prepared with PyMOL v2.3.0.

Target **T160** consisted of the surface assembly domain of SAP, the surface layer protein from *B. anthracis* . The assembly domain folded into six discrete structural domains and was solved in complex with two (different) antibodies to high resolution by X‐ray diffraction. The structure (now published as PDB ID 6HHU) [23] was offered to CAPRI by *Han Remaut* and *Antonella Fioravanti* from the VIB‐VUB Center for Structural Biology in Brussels, Belgium. To facilitate the modeling task, the atomic backbone coordinates of the individual domains D1—D6 in random orientation and translation were supplied. The prediction challenge consisted of modeling the 3D structure of the SAP assembly domain, that is, the intramolecular contacts between the SAP domains and the conformations of the connecting loops, as well as the complex with the two nanobodies.

The two targets **T161** and **T162** from Round 48 stem from the same biological system, namely the *E. coli* toxin‐antitoxin complex, RnlA‐RnlB. T161 consists of the unbound form of the toxin, whereas T162 was the homo‐dimer of the toxin protein bound to two antitoxin molecules. It was offered to CAPRI by *Remy Loris* and *Gabriela Garcia‐Rodriguez* from the VIB‐VUB in Brussels, Belgium, and is now published as PDB ID 6Y2P [24]. In the complex, the toxin homodimer forms a completely different dimer than in its unbound form. The challenge of T161 was to predict the bound form of the toxin homodimer; the challenge of T162 was to predict the structure of the full toxin‐antitoxin complex.

Target **T163** is a heterocomplex with A2B2 stoichiometry, consisting of two small helical protein components SYCE2 and TEX12 of the synaptonemal complex. Offered by *Owen Davies* from Newcastle University, UK, now published as PDB ID 6R17 [25].

Target **T186** is a cryo‐EM structure, representing a large protein complex that is part of the peripheral arm of the *E. coli* respiratory complex NADH:ubiquitine oxidoreductase. It consists of six polypeptides, one of which has a *ca*. 100 residue loop that is bound to a large pocket of the complex. The loop adopts a well‐defined conformation with little secondary structure elements. An existing template from a related complex from *T. thermophilus* lacks the 100‐residue loop. The challenge of this target was to correctly model the bound conformation of the loop.

The first “post‐AlphaFold” targets were **T187** and **T188**, which both featured the same bacterial ( *B. thuringiensis* ) protein‐DNA complex, representing a transposase homodimer in complex with 2 dsDNA fragments. Both targets required the prediction of the full complex, but for T188 the bound conformation of a single dsDNA fragment was supplied. The bound transposase dimer features a significant conformational change with respect to its unbound dimeric structure. Modeling this conformational change was considered a near‐impossibility in “pre‐AlphaFold” times, but AlphaFold was found to predict the DNA‐bound conformation of the transposase monomer. In addition, the DNA itself showed strong bending; hence for T188 a single dsDNA structure in its bound conformation was supplied. Targets T186 and T187/T188 were provided by *Rouslan Efremov* from VIB‐VUB Brussels, Belgium, and are now published as PDB ID 7NZ1 and 7QD5, respectively [26, 27].

Lastly, Round 55 featured four targets. Target **T231** was an antibody‐peptide target provided by *Matti Pronker* and *Bert Janssen* from Utrecht University, The Netherlands, and is now published as PDB ID 8RMO [28]. Target **T232**, featuring a phosphatase‐regulator complex, was provided by *Wei Huang* from Case Western Reserve University, USA, and is not yet published. A low‐resolution crystal structure of the same complex existed, but it was not found to fit the EM density map. Targets **T233** and **T234**, finally, were two antibodies bound to the human Major Histocompatibility Complex‐I. They were offered to CAPRI by *Josan Marquez* from EMBL Grenoble, France, and are also not yet published.

## 3 Participation Statistics

A total of 64 groups participated in the six CAPRI Rounds evaluated here. These can be categorized into 61 predictor and 53 scorer groups, with a significant overlap between the two. Table 2 lists the number of registered groups (A) and the number of submitted models (B), with a subdivision into pre‐ and post‐AlphaFold Rounds.

**TABLE 2:** CAPRI 8th Evaluation period participation statistics.

	Overall			Pre‐AlphaFold		Post‐AlphaFold	
	Total	Human	Server	Human	Server	Human	Server
(A) Number of groups							
Predictors	61	50	11	40	9	31	9
Scorers	53	43	10	38	8	23	7
All	64	53	11	45	9	32	9
(B) Number of models							
Predictors	1329			629		700	
Scorers	1640			825		815	
Total	18 542			8535		10 007	

*Note:* (A) Number of registered predictor and scorer groups, separated into human and server groups. Note that the same group (identified by their PI name) can participate both pre‐ and post‐AlphaFold, and can be both predictor and scorer. (B) Number of evaluated models for the predictor groups (max. 5 submitted models), Scorer groups (max. 10 submitted models) and the total, which includes the predictor models (max. 100 submitted models) that go into the scoring experiment.

A pronounced drop in registration can be observed between pre‐ and post‐AlphaFold Rounds. This decrease is even more significant if one considers that 10 of the registered groups (8 human, 2 server) only registered for the post‐AlphaFold Rounds.

In terms of submitted models, it should be noted that not all registered groups participate in all targets; typically, a target attracts between 20 and 30 predictor groups and between 15 and 20 scorer groups, resulting in a collection of 100–150 predictor models and 150–200 scorer models. Interestingly, these participation statistics do not change between pre‐ and post‐AlphaFold Rounds.

We also note that the total number of submitted models is balanced between pre‐ and post‐AlphaFold rounds, which, together with the lower number of registrants, shows an increase in the number of registered groups that actually submit models. The higher total number of models, which includes the predictor models that go into the Scoring Experiment (see caption of Table 2), is consistent with this analysis.

## 4 Assessment Criteria and Performance Ranking

The classical CAPRI evaluation and ranking protocols were used to assess the quality of submitted models for most targets [7, 29, 30]. The protocol was modified for the antibody‐peptide target T231 of Round 55 as previously to account for the smaller interaction surface [11]. Briefly, to assess the quality of modeled complexes, the native contact recall (*f*_nat_) was calculated on residue‐residue contacts using a 5 Å distance threshold (4 Å for antibody‐peptide). Two residues are considered to be in contact if any of their heavy atoms are within the defined distance cutoff of one another. This metric is complemented by two rmsd‐based quantities: the interface rmsd *i*‐rms (residues forming the interface are defined using a 10 Å distance threshold (8 Å for antibody‐peptide)), and the ligand misorientation rmsd *L*‐rms, calculated over the ligand main‐chain atoms after optimally superimposing the receptor entities, with the receptor defined as the larger entity of the binding partners. Additional metrics are calculated as well, but these are not used to rank predictions.

The CAPRI assessment is interface centric. Individual interfaces are each assessed separately, with selected combination operators applied to assess the quality of a target as a whole. We use “target” to refer to any ground‐truth structure submitted for blind prediction. When a target is too large to be evaluated as a single unit, we split it into smaller subtargets, each itself a target. For the purpose of ranking groups, multiple interfaces from larger targets may be grouped into these subtargets to avoid unduly penalizing performance on especially difficult to predict interfaces. These (sub)‐targets were previously called “Assessment Units”, a term we decided to deprecate because it conflates the act of judging with the items being judged and fails to convey their relationship to the original targets; so we now use simply target (whole or fragment) and subtarget. When a target is split into subtargets, the target as a whole is never considered in the ranking.

For three pre‐AlphaFold targets, this was the case. T163—a 2 by 2 association of long alpha‐helices—was split into three subtargets: T163.2 and T163.3 counted the best of the two homomeric and three heteromeric interfaces, respectively, whereas T163.1 model quality was determined through the average of all five interfaces. T186—the loop modeling target—was also split into three subtargets: T186.1, comprising the interface between chains G (including the loop) and D; T186.2, assessing the intrachain interface between the loop and the rest of chain G; and T186.3, which was limited to the interface between the loop and chain D. Finally, T160, which counted no less than 8 different interfaces between the domains 1–6 and the two nanobodies, was split into as many subtargets: T160.1 and T160.2 for the interaction with the two nanobodies; T160.3–7 for each consecutive domain triple (D1,2,3—D2,3,4—D3,4,5—D4,5,6 and D5,6,3); and T168.8 as the best of all 6 domain‐domain interfaces.

### 4.1 Performance Ranking

The performance of predictors, servers, and scorers was ranked as previously described [11]. Briefly, the global score, Score_G, is calculated as follows:

$$\text{Score}\_G=\omega_{1}\;N_{\mathrm{ACC}}+\omega_{2}\;N_{\mathrm{MED}}+\omega_{3}\;N_{\text{HIGH}}$$

Here, *N* represents the number of targets for which at least an acceptable, medium, or high‐quality model was obtained. The weighting factors ω were taken as 1, 2, and 3, resp.

## 5 Prediction Results

In the following, we summarize the prediction results for the different targets in chronological order and present the global performance ranking across groups and automatic servers. The performance of predictors and servers was ranked on the basis of the best model in the top five submissions for each target; for scorers, this was the top 10 submissions for each target. A full account of all the results for these targets is available at the official CAPRI website (https://www.capri‐docking.org/assessment). The results are split into the pre‐ and post‐AlphaFold era. For an overview of the CAPRI timeline, we refer to Figure 2.

**FIGURE 2:**
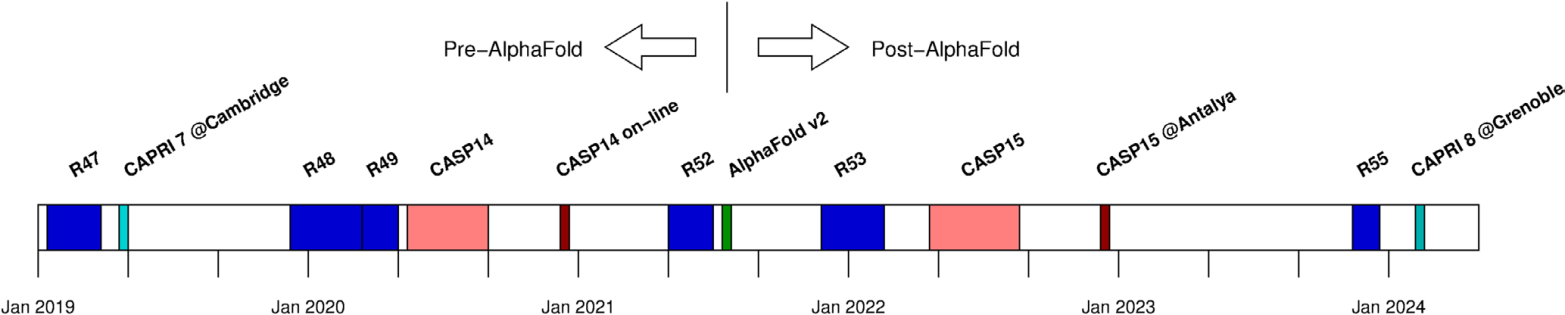
The timeline of the 8th CAPRI Evaluation period. CAPRI Rounds and Meetings are indicated by blue and cyan boxes, resp. CASP Rounds and Meetings are indicated by pink and brown boxes, resp. The release of AlphaFold v2 is indicated by the green box, separating the timeline in a pre‐ and post‐AlphaFold period.

### 5.1 Results on the Pre‐AlphaFold Targets

**T160**, the surface layer assembly domain of *B. anthracis* , was resolved in complex with two nanobodies by X‐ray crystallography at 2.7 Å resolution. This turned out to be a difficult target in all aspects: only three of the six inter‐domain interfaces were predicted by more than one predictor group, and none of the predictors nor scorers managed to generate or identify any correct model for the nanobody interfaces. The S‐layer domain assembles in a planar S shape, with the C‐terminal D6 domain curving back onto the structure, creating an extra interdomain interface (see Figure 3A). The largest interfaces between consecutive domains—interfaces 3 and 6—were, together with interface 8, the best predicted. Medium‐quality submissions were made by the groups of Andreani/Guerois, Kozakov/Vajda, and Venclovas. Acceptable models were, in addition to these groups, created by Fernandez‐Recio and the GalaxyPPDock server. Gray produced an acceptable model for interface 7. With so few acceptable or better models in the set, singling out the good models was a challenging task for the scorers. Venclovas correctly identified the best model by Andreani/Guerois and obtained medium quality for interfaces 3 and 8. This medium‐quality model is depicted in Figure 3B. Fernandez‐Recio, Kihara, Venclovas, Bates, and the server LZerD achieved acceptable quality for interface 6, whereas the server HDOCK managed an acceptable‐quality model for the smallest interface, interface 9. The target split‐up resulted in a final score of 5/1** for Andreani/Guerois, 3/1** for Kozakov/Vajda and Venclovas, and one acceptable target for Fernandez‐Recio, Gray, and the GalaxyPPDock server. For scorers, Venclovas had 5/1**, and Kihara, Bates, and the LZerD and HDOCK servers had one acceptable target.

**FIGURE 3:**
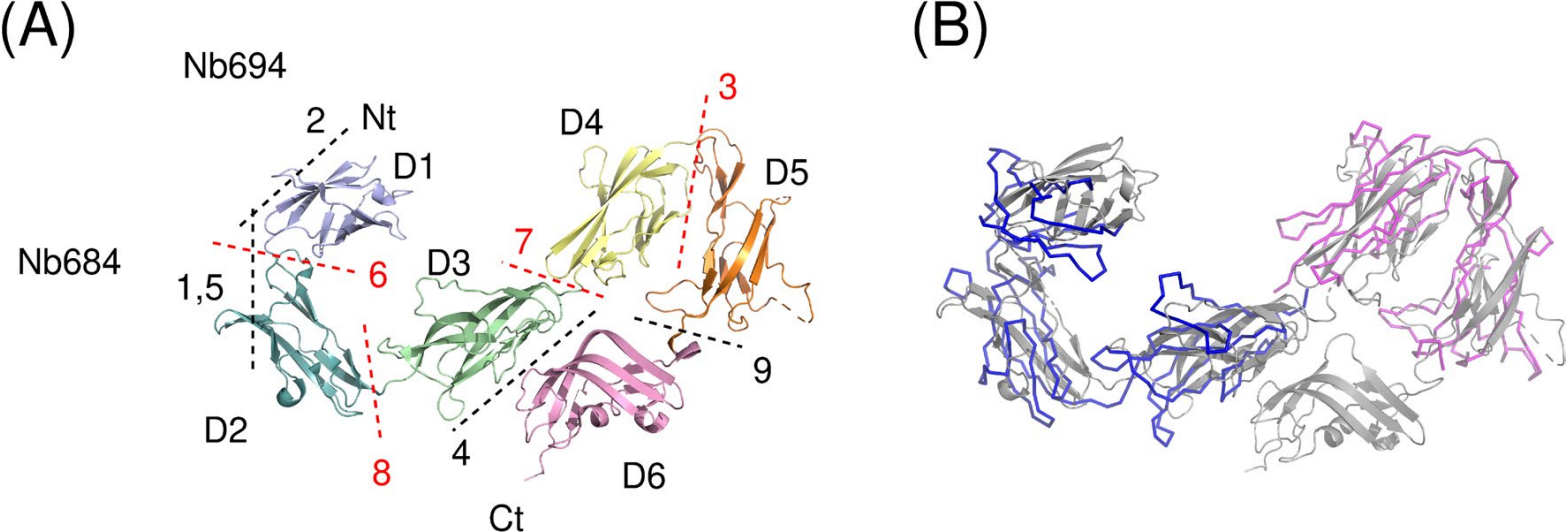
Organization of the B. anthracis surface layer assembly domain, T160. (A) Organization of domains D1—D6. The two nanobodies are not depicted, as they lie on top, perpendicular to the plane of the molecule; Nb694 interacts with D1 through interface 2, and Nb684 interacts with D1 and D2 through interfaces 1 and 5. The inter‐domain surfaces are indicated with dashed lines and numbered in decreasing size, going from 610 Å^2^ for interface 1 to 150 Å^2^ for interface 9. Accurately predicted interfaces (by any group) are shown in red. (B) Model 2 of the Andreani/Guerois group overlapped onto the target. This was the only model accurately predicting three of the nine interfaces: Interfaces 3 and 8 to medium quality, and interface 6 to acceptable quality. The model is depicted in blue and purple to account for the fact that interface 7 was wrongly predicted.

The two targets **T161** and **T162** of Round 48 feature the same protein complex: the RnlA‐RnlB toxin‐antitoxin complex. The difficulty in this system was a conformational change of the RnlA dimer upon binding of RnlB. The longer RnlA toxin consists of three domains: D1, D2, and D3. The unbound RnlA homodimer associates through D3. Upon binding of two RnlB antitoxin molecules, the bound RnlA homodimer shows a conformational rearrangement for D1 and D2, forming an intertwined dimer through D2 and D3. Each D3 domain binds one RnlB molecule. D1 could therefore be ignored for both targets; the information that was shared with the predictors was to focus on D3, know that D2 moves, and ignore D1. For **T161**, the bound RnlA dimer, none of the predictors foresaw the D2 intertwining. However, Bates managed—as the only predictor—to generate an acceptable model for the dimer through a correct association of the two D3 domains. For **T162**, which could be reduced to the binding of RnlB to RnlA/D3, all submitted models (1756 predictor and 165 scorer models; totaling 1921 structures) were made available to predictors. The poor performance on T161 may have impacted the results on T162, as only one team—Andreani/Guerois—managed to produce correct models, with one of them of medium quality. This model was not based on any of the submitted models for T161. No acceptable scorer submissions were made for these two targets.

**T163** of Round 49 featured a 2:2 association of two helical protein components, SYCE2 and TEX12. An unbound dimeric structure of TEX12 could be identified, but its binding mode differs from its association in T163. The largest interface between the two SYCE2 molecules buries 1400 Å^2^. The smallest interface, number 5, between the two TEX12 molecules buries a third of that. The other three interfaces are heteromeric and bury between 900 and 1200 Å^2^. The largest heteromeric interface was the only interface correctly predicted by three predictor and three scorer groups. All other interfaces were correctly predicted by at most one predictor or scorer group; with the notable exception of Kihara—nobody managed to correctly predict two interfaces. As one of the interfaces was heteromeric (T163.3) and the other homomeric (T163.2), Kihara achieved 2/1** on this target, while Venclovas, Kihara, Gray, and the LZerD server all had acceptable models. Scorers Seok and Kihara both identified an interface of medium quality, and Fernandez‐Recio, Oliva, and Perthold of acceptable quality.

**T186** featured the oxidoreductase complex of the respiratory complex peripheral arm of *E. coli* . A template from *T. thermophilus* lacking the ~100 residue loop to be modeled was provided. The loop was part of the chain G sequence and buried 2675 Å^2^ with the remainder of chain G (T186.2), and 1070 Å^2^ with chain D (T186.3). This was yet again an extremely challenging target, with no acceptable solutions for T186.3, and only one predictor (Kihara) and 2 scorer groups (Kihara and Venclovas) obtaining acceptable quality models for T186.2. Significantly more groups (five predictors and 12 scorers including three scoring servers) achieved acceptable quality models for T186.1, the interface between chains G and D, but the fact that a template was available, and no medium quality model was obtained illustrates the poor quality of the loop modeling.

### 5.2 Results on the Post‐AlphaFold Targets

The first targets of the post‐AlphaFold era would not have been proposed as CAPRI targets had AlphaFold not existed. **T187** and **T188** feature the same complex, a transposase homodimer bound to two double‐stranded DNA molecules. An unbound structure of the transposase homodimer existed, but it would not have been able to accommodate the dsDNA, making the modeling a virtually impossible task. However, upon running AlphaFold, we discovered that it was modeling the *bound* state of the monomer. At the time, it was not evident to run multimers with AlphaFold. As such, most predictors used classical docking to create the dimer, and then protein‐DNA docking to create the full complex. Modeling a straight double‐stranded helix would not allow capturing the majority of the protein‐DNA contacts properly, explaining the fact that no acceptable solutions were obtained for T187. Providing a single dsDNA molecule in bound conformation for T188 removed the requirement of modeling the correct conformation of the DNA. Still, only two predictor groups managed to produce acceptable models: Fernandez‐Recio and Pierce. It should be noted that Fernandez‐Recio did not produce an acceptable protein dimer model based on CAPRI criteria for the protein–protein interface, but did reproduce the protein‐DNA contacts quite accurately, resulting in a final model with *f*_nat_ = 0.338 and *i*‐rms = 4.0 Å. Pierce, however, did produce an acceptable dimer model, resulting in a better final model, with *f*_nat_ = 0.405 and *i*‐rms = 3.3 Å. Following our expectations, producing an acceptable transposase dimer was possible, although not a given, as illustrated in Figure 4. Many acceptable and medium‐quality protein dimers were created, having *i*‐rms roughly between 2 and 4 Å, but the modeling of the protein‐DNA interface ranged between 3 and more than 60 Å. Five models are worth noting: the three already mentioned acceptable models for T188, and two incorrect models for T187 (one with medium quality protein dimer interface), both by Kihara. These models are the five left‐most points in Figure 4.

**FIGURE 4:**
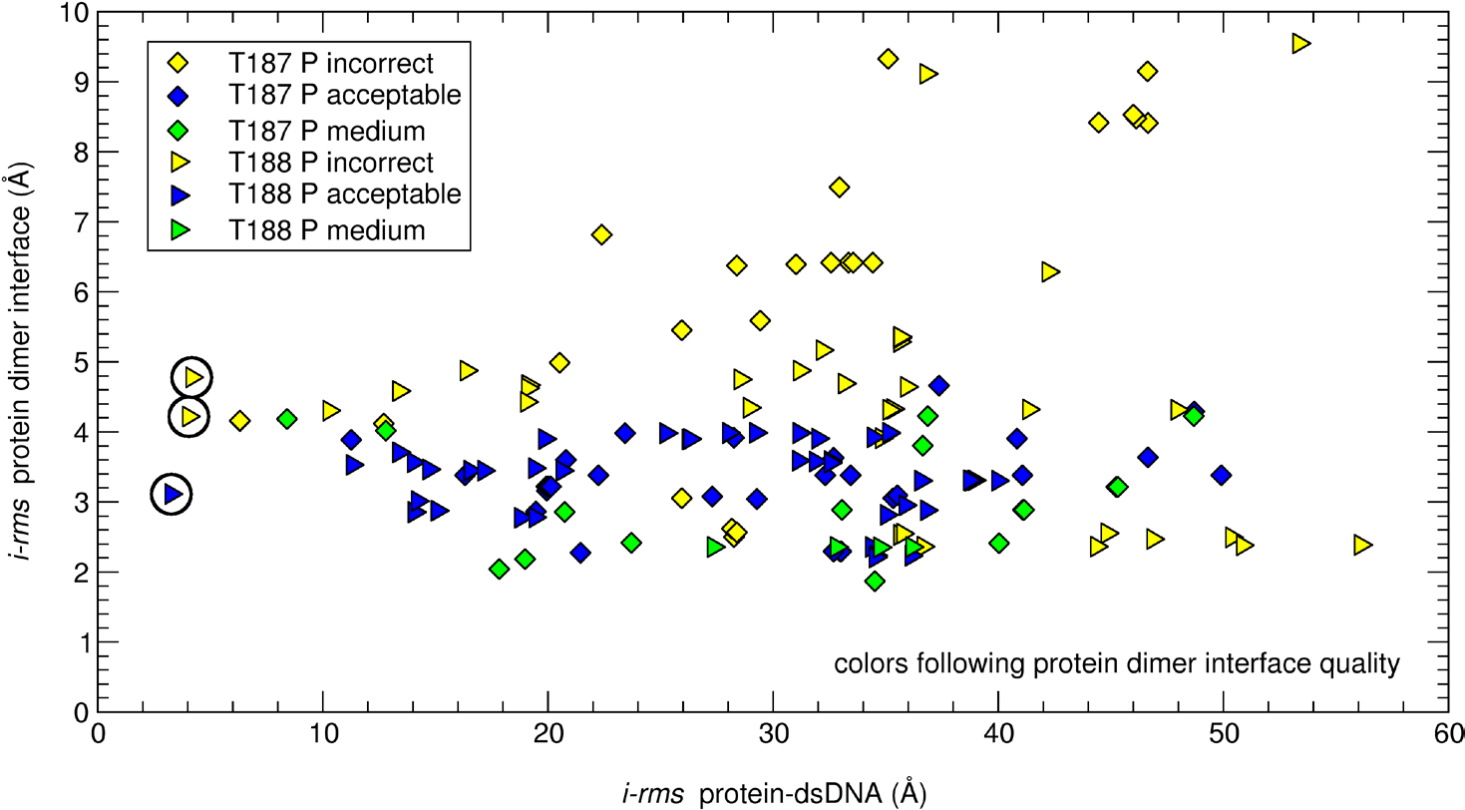
Combined interface quality of targets T187 and T188. The figure shows for the top five predictor models submitted to T187 and T188 the interface quality as expressed by the *i*‐rms for the protein–protein (*y*‐axis) and protein‐dsDNA (*x*‐axis) interfaces. T187 submissions are indicated by diamonds and T188 by right‐handed triangles. The color of the icons reflects the CAPRI quality of the protein dimer interface. Note that only the protein‐DNA interface was part of the assessment and not the protein dimer interface. The only three acceptable models for T188 are encircled.

Round 55 represents the first true post‐AlphaFold CAPRI Round, in the sense that by now all participants had incorporated AlphaFold in one way or another into their pipelines. For all targets in this Round, the default 25 models as calculated by AlphaFold v2.3 were made available to predictors. The only “conventional” target in that Round was **T232**, an enzyme/inhibitor complex featuring a serine/threonine phosphatase in complex with its regulator TIPRL. A 3.8 Å crystal structure of the complex existed (PDB ID 5W0W) [31], which was not compatible with the EM density map of T232. This information was shared with the participants, but the density map was not. The phosphatase consists of two chains, A and C, and in the crystal structure, TIPRL makes contact with both chains. In the EM structure of T232, TIPRL is only in contact with chain C, burying 1000 Å^2^. It is displaced by 25.9 Å and rotated over 129°, resulting in a completely different interface, as indicated by the *i*‐rms vs. the crystal structure of 18.6 Å. This was, however, not a difficult target: all 25 AlphaFold models were of medium quality. No less than 24 of the 25 predictor groups also submitted medium quality models for this target, as did all 14 scorer groups. These included 5 docking servers and 3 scoring servers. Interestingly, virtually no lower quality, acceptable models were submitted.

The other three targets of the Round were antibodies. **T231** featured an antibody binding to a short peptide fragment, requiring the application of the more stringent criteria developed for protein‐peptide assessment [10, 11]. The pre‐calculated AlphaFold models were mostly of acceptable quality, with two medium‐quality (ranked 14 and 15) and one incorrect model (ranked 22). Medium‐quality models were submitted by 10 predictor groups, among which three servers, with an additional 8 groups submitting models of acceptable quality. Of the 14 scorer groups, 6 submitted medium‐quality models, and another 7 submitted acceptable‐quality models. The best submitted model, shown in Figure 5, came from the Kozakov/Vajda group: it was their first‐ranked model and featured a native contact recall (*f*_nat_) of 82.6%, with a *L*‐rms of 1.28 Å and *i*‐rms of 0.71 Å. This model was recognized by the HDOCK scoring server and ranked 6 in their submission.

**FIGURE 5:**
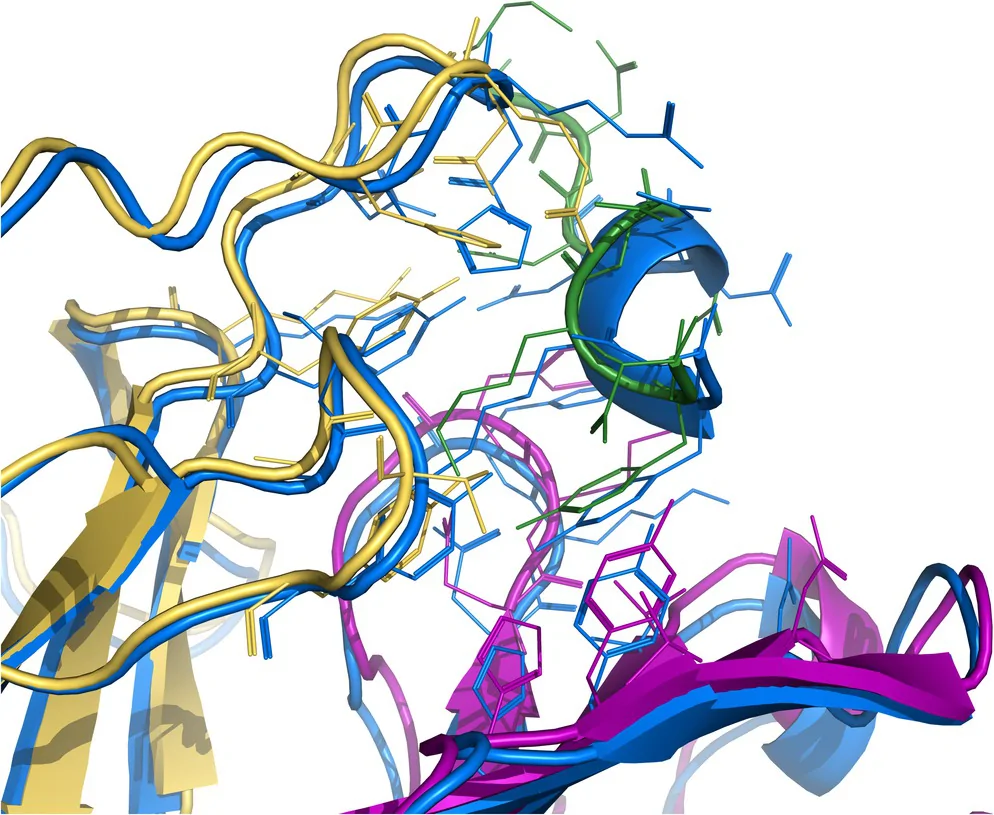
Best model for the antibody‐peptide structure T231. Superimposition of the best prediction for T231 onto the reference structure. The structures are colored as follows: Magenta for the antibody heavy chain, yellow for the light chain, and green for the bound peptide; the reference structure is colored blue. The model was submitted by the Kozakov/Vajda team as first ranked.

Targets **T233** and **T234** constitute the final targets of this assessment period. Both targets feature binding of an antibody to the human major histocompatibility complex‐I: HLA‐A*11:01 for T233 and HLA‐A*02:01 for T234. As for all these systems, a natural peptide was found in the HLA pocket, but its sequence could not be determined, and it was not relevant for the interactions. As such, its presence was not disclosed to the predictors, an information that could have been relevant for the modeling from the a posteriori feedback we received. This was a difficult target, as indicated by the 25 pre‐calculated AlphaFold structures, which were all incorrect. Nonetheless, 6 predictor groups (Kihara, Zou, Venclovas, S.Chang, Huang, and server HDOCK) all managed to obtain medium‐quality predictions for T233. No acceptable submissions were recorded for T234.

In such cases, when few acceptable models are produced, the CAPRI Scoring Experiment is particularly motivating. Whereas only the first five submitted models are included for the participant ranking, all submitted models (up to 100) are included in the Scoring Experiment, and all submitted models are assessed. The models are shuffled and anonymized and provided to the scorers. For T233, this resulted in a set of 1696 models, of which 7 were of acceptable quality, 18 of medium quality, and a single one was of high quality. Therefore, barely 1.5% of models in the scoring set were of acceptable quality or better. Six scorer groups (Fernandez‐Recio, Zou, Venclovas, S.Chang, Huang, and server HDOCK) managed to recognize a medium‐quality model. The high‐quality model in the set, submitted by Pierce as model 34, with *f*_nat_ = 0.776, *L*‐rms = 3.26 Å, and *i*‐rms = 0.94 Å, was however not recognized. While there were no predictor‐submitted models of acceptable quality or better for T234, there was one acceptable model submitted by scorer Giulini. This was one out of only four “correct” models in the scorer set (three acceptable and one medium), out of a total of 1677 models, corresponding to a mere 0.24%. The medium‐quality model in the set, submitted by Venclovas as ranked 48th (*f*_nat_ = 0.818, *L*‐rms = 18.26 Å, *i*‐rms = 1.18 Å), was not identified by any of the scorers.

### 5.3 AlphaFold Performance

On July 15th, 2021, AlphaFold v2 was published with open source code [13]. Less than 2 weeks later, the DeepMind and ColabFold notebooks ensured global accessibility [32]. Rounds 47–49 and 52 of this Evaluation period were held before the release of AlphaFold, and Rounds 53 and 55 were held after. While the first AlphaFold‐Multimer version (v2.1) became available on Oct 5th, 2021, it was plagued by clashes and unrealistic protein structures. An updated version, v2.2, was released 6 months later, and by the time Round 55 was organized, all predictors had fully integrated AlphaFold into their respective pipelines. It was for obvious reasons not used before R53, whereas for R53, AlphaFold was used “as is”, often only in monomer mode.

Both AlphaFold v2.1 and v2.2 were parameterized on the PDB data set up to April 2018. Since all targets of this evaluation period were released afterwards, they fall out of the training set and could therefore be predicted with AlphaFold, which we did. In order to avoid introducing any bias due to template availability, the template date limit for any target was set to the first of the month that the respective target was released, for example, for T160, released on Aug 29th, 2018, the template date limit was set to Aug 1st, 2018. All other settings were left to default, and no relaxation was performed. The AlphaFold prediction results are listed in Table 3. Overall, it can be seen that for the pre‐AlphaFold targets, AlphaFold does remarkably well, globally improving the prediction quality over the best predictors by one model quality category, but only for those interfaces where at least an acceptable level was reached. The notable exception is T163, where—for all five interfaces collectively—AlphaFold produces a medium‐quality model; the clashes for the second interface are only just above the clash threshold and would likely be solved with relaxation. A remarkable performance can be observed for target T186 in modeling the ~100‐residue loop, as AlphaFold achieves a high‐quality prediction, with rms values below 1 Å. A detailed view of the loop, including the chains of the AlphaFold model superimposed onto the target, is shown in Figure 6. For target T160, the organization of the surface layer assembly domain, near‐native structures of the linkages between domains D1, D2, and D3, and between domain D4 and D5, are obtained. However, as for the conventional predictors, all nanobody binding models were incorrect (interface 2 for Nb694, and interfaces 1 and 5 for Nb684). The difficulty for AlphaFold in predicting nanobody and antibody binding has been observed before [21]. The poor performance on the three antibody targets of post‐AlphaFold Round 55 is therefore not surprising, but it should be noted that the best predictor groups did produce medium quality models.

**TABLE 3:** AlphaFold performance on the 8th Evaluation period CAPRI targets.

Target	#models produced	AF versions	Interface	Best predictors	AlphaFold
Pre‐AlphaFold targets					
T160	25	v2.1	3 & 8	Medium	High
			6	Acceptable	Medium
			7	Acceptable	Incorrect
			Rest	Incorrect	Incorrect
T161	50	v2.1, v2.2		Acceptable	Medium
T162	50	v2.1, v2.2		Medium	High
T163	50	v2.1, v2.2	1	Incorrect	Medium
			2	Acceptable	Incorrect (+4 × medium with clashes)
			3	Incorrect	High
			4	Acceptable	Medium
			5	Medium	Medium
T186	25	v2.1		Acceptable	High
Post‐AlphaFold targets					
T187^a^	50	v2.1, v2.2		Medium	Medium
T231	25	v2.3		Medium	Acceptable
T232	25	v2.3		Medium	Medium
T233	25	v2.3		Medium	Incorrect
T234	25	v2.3		Incorrect	Incorrect

*Note:* Targets are listed in chronological order. For each listed AF version, 25 models were created; the top‐5 following confidence rankings were evaluated using the exact same CAPRI criteria as for predictor and scorer submissions.

^a^ Protein–protein interface only.

**FIGURE 6:**
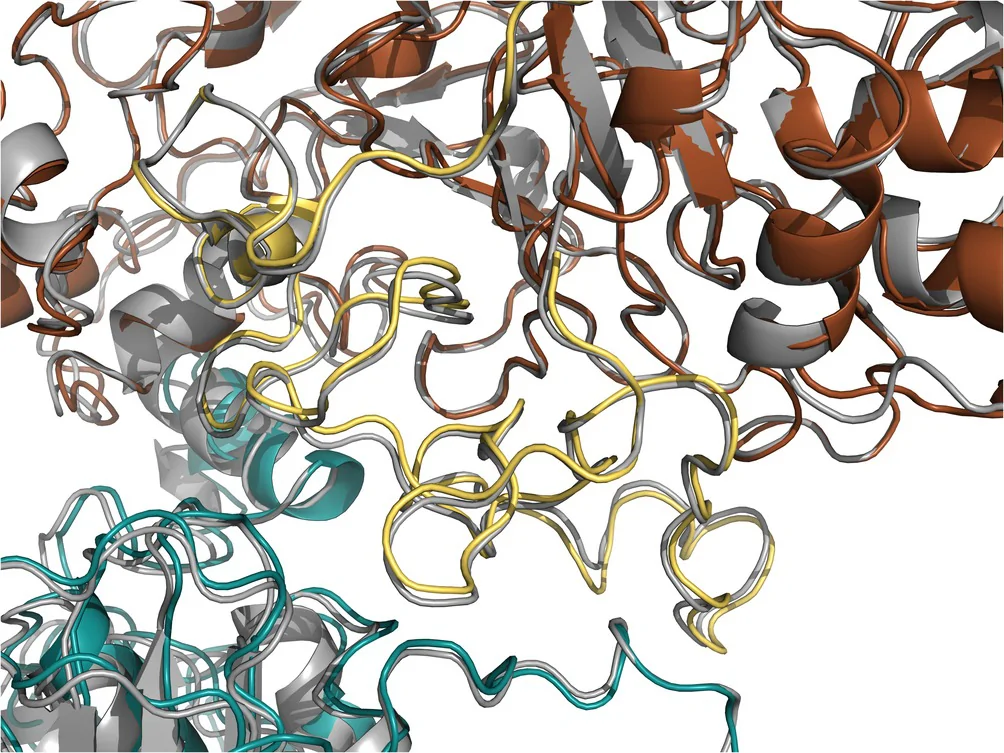
AlphaFold model for the loop in T186. The reference structure is colored as follows: Chain D, blue; chain G, brown; the loop, yellow. The AlphaFold model (ranked 1) is superimposed onto the reference structure and colored gray. This model is of high quality, that is, both *L*‐rms and *i*‐rms values are below 1 Å.

## 6 Performance of Predictors, Servers, and Scorers

Groups (predictors, servers, and scorers) were ranked according to their prediction performance over the 10 targets evaluated here (21 (sub)‐targets). This ranking represents the official performance ranking for the 8th Evaluation period, comprising Rounds 47–49, 52, 53, and 55. The ranking includes contributions from both the pre‐ and post‐AlphaFold Rounds, which are listed separately. In addition, we also discuss the performance of vanilla AlphaFold. For a full account of the results obtained by each group, we refer the reader to the CAPRI web site (https://www.capri‐docking.org/assessment/).

### 6.1 Performance of Predictor Groups

The consolidated ranking of predictor groups (including servers) is shown in Table 4A. The ranking shows the four best performing groups to be those of Venclovas, Kihara, and the teams Andreani/Guerois and Kozakov/Vajda, with correct predictions for at least 5 targets. One other group—Fernandez‐Recio—shows correct predictions for 5 targets, but only one of those is of medium quality. Several observations can be made. The four mentioned groups are the only groups that managed to obtain medium‐quality models for the pre‐AlphaFold targets, whereas most groups submitted medium‐quality models for one or even several of the post‐AlphaFold targets. Of these four, the performance of the Andreani/Guerois team is remarkable, since they have not participated in any of the post‐AlphaFold targets. Had they done so and only submitted default AlphaFold models (see the following subsection), they would have shared the top position with the Venclovas team.

**TABLE 4:** Predictor and scorer performance.

Rank	Group name	#targets	Top 5	Pre‐AlphaFold		Post‐AlphaFold	
			Performance	Rank	Performance	Rank	Performance
(A) Predictor group ranking (human and server)							
1	Venclovas	20	7/4**	2	4/1**	1	3**
2	Kihara	21	7/3**	2	4/1**	5	3/2**
3	Andreani/Guerois	13	6/2**	1	6/2**	—	—
4	Kozakov/Vajda	20	5/3**	4	3/1**	8	2**
5	Huang	21	4/3**	7	1	1	3**
6	Fernandez‐Recio	21	5/1**	5	2	8	3/1**
7	Zou, HDOCK	21	3**	—	0	1	3**
8	S.Chang	18	4/2**	7	1	5	3/2**
9	LZERD	21	3/2**	7	1	8	2**
10	Pierce	18	3/2**	—	0	5	3/2**
11	Vakser	17	2**	—	0	8	2**
12	Gray	17	3/1**	5	2	18	1**
13	CLUSPRO	19	2**	—	0	8	2**
14	Bates	13	3/1**	7	1	14	2/1**
15	Furman	6	3/1**	7	1	14	2/1**
16	Karaca	4	2**	—	—	8	2**
17	Brysbaert, Giulini	4	2/1**	—	—	14	2/1**
(B) Server group ranking							
1	HDOCK	21	3**	—	0	1	3**
2	LZERD	21	3/2**	1	1	2	2**
3	CLUSPRO	19	2**	—	0	2	2**
4	HADDOCK	18	1**	—	0	4	1**
5	MDOCKPP	21	1**	—	0	4	1**
6	GALAXYPPDOCK	13	1	1	1	—	—
7	PYDOCKWEB	9	1	—	0	6	1
(C) Scorer group ranking (human and servers)							
1	Venclovas	21	11/3**	1	8/1**	6	3/2**
2	Huang	21	8/4**	2	5/1**	2	3**
3	Fernandez‐Recio	21	7/3**	4	3/1**	2	4/2**
4	Kihara	21	7/2**	3	4/1**	8	3/1**
5	Zou	21	5/3**	9	1	1	4/3**
7	HDOCK	21	5/3**	6	2	2	3**
8	S.Chang	10	4/3**	9	1	2	3**
9	Oliva	20	4/2**	6	2	8	2**
10	LZERD	21	5/1**	5	3	11	2/1**
11	MDOCKPP	21	4/1**	9	1	8	3/1**
12	Giulini	4	3/2**	—	0	6	3/2**
13	Bates	21	3/1**	9	1	11	2/1**

*Note:* (A) Predictor groups, human and servers combined; (B) automatic servers; (C) scorer groups, human and servers combined. The lists are restricted to those groups producing at least acceptable models for two—(A) and (C)—or one—(B)—target, resp. For all groups, the model of highest quality in their top‐5 submission (top‐10 for scorers) for every target is considered, and ranking is performed following the formula for Score_G as outlined in the text. Groups are listed by the name of the corresponding PI. Servers are listed by their acronym. The performance is listed by the number of targets for which a model of acceptable quality or better was produced, followed by the number of these models that were of medium (**) quality. A dash indicates that no submission was made.

Focusing rather on the real‐life post‐AlphaFold targets, no less than eight groups managed to predict three targets to acceptable quality or better. Venclovas, Zou, and Huang and his server HDOCK predicted all of these to medium quality and would rank therefore *ex aequo* at first position. The other four groups—Kihara, S. Chang, Pierce, and Fernandez‐Recio—had models of acceptable quality in the set. It should be noted that Pierce and Fernandez‐Recio were the only predictors with acceptable models for the difficult target T187/T188, capitalizing on their expertise in protein‐DNA modeling.

### 6.2 Performance of AlphaFold

Translating the per‐interface prediction performance of AlphaFold (as shown in Table 3) to targets results in a ranking performance of 14/5***/4**. This, compared to the best performance of 7/4** by the group of Venclovas, would seem to indicate that AlphaFold largely outperforms all predictor groups. However, this image is heavily skewed. Dividing the contribution into a pre‐ and post‐AlphaFold contribution shows that their overall performance is largely due to the exceptional models that were produced for the pre‐AlphaFold targets, resulting in a 12/5***/3** performance, whereas for the post‐AlphaFold targets only 2/1** is obtained, bearing in mind that the protein dimer interface of T187 was not a target. Comparing the AlphaFold performance on the pre‐AlphaFold targets to that of the best predictors, it is obvious that AlphaFold generated far better models.

Prior to the public release of AlphaFold, the CASP14/CAPRI prediction Round was held, with results presented at the on‐line CASP14 meeting [22]. Targets presented there were also not part of the AlphaFold training data, so we can apply vanilla AlphaFold to that set as well, and compare to the results obtained here. The score of the best predictor then—Seok—was 9/4**, that is, 9 targets were correctly predicted, of which four were of medium quality. Seok was the only predictor with 9 correct targets, but without any of them being of high quality. Other predictors did have high‐quality targets, but none of them had more than one. Applying AlphaFold v2.1 to the CASP14/CAPRI target set, we obtain a score of 9/4***/1**, that is, the same 9 correct targets that Seok obtained, but with superior quality as four of these targets were of high quality. In our report, we already noted the extraordinary capability of AlphaFold to generate the correct structure of protein monomers in their oligomeric assembly state [22, 33].

### 6.3 Performance of Scorer Groups

The ranking of scorer groups is shown in Table 4C. Similar to the predictor performance, the list is topped by four predictor groups that managed medium‐quality models for both pre‐ and post‐AlphaFold targets. These are Venclovas, Huang, Fernandez‐Recio, and Kihara. Venclovas is the top scorer group (they were also the top predictor group). Fernandez‐Recio and Zou were the only scorer groups with models of acceptable quality or better for 4 of the 6 post‐AlphaFold targets, one of them being T188, the protein‐DNA target.

Most scorers seem to do significantly better at scoring than predicting; Zou has 5/3** as a scorer, but only 3** as a predictor, Fernandez‐Recio lists 7/3** as a scorer, but only 5/1** as a predictor, and top scorer and predictor Venclovas scores 11/3** and 7/4**, resp. This apparent discrepancy is only due in part to the fact that scorer ranking considers the top‐10 submissions, as opposed to top‐5 for predictors. Scorer Venclovas, for instance, would still rank first—with 10/3**—if only the top‐5 were taken into consideration, but would fall to third position for top‐1 ranking, scoring only 2**. In contrast to this, predictor Venclovas ranks first in all three cases, with 7/4** for both top‐10 and top‐5, and 6/3** for top‐1. The Scoring Experiment in CAPRI, originally suggested at the CAPRI meeting in Gaeta, Italy in 2004, and implemented for the third CAPRI meeting in Toronto, Canada, significantly enriches in correct structures the set of structures one can choose from [7, 8]. Paradoxically though, this makes identifying good models easier, but identifying the *best* models more difficult.

Figure 7 shows the rank consistency for the top nine predictors and scorers. The figure shows the scaled ranksum (the average rank based on top‐1, top‐5 and top‐10 submissions) as a function of actual rank, which—for the purpose of comparison—is calculated on the top‐5 submissions for both predictors and scorers. The figure shows a close to ideal linear behavior for predictors; for example, Venclovas, Kihara, and Andreani/Guerois are ranked first, second, and third, but are also ranked exactly first, second, and third on the average ranking. In contrast to this, *scorers* Venclovas and Kihara do slightly worse on the average ranking: any deviation from the diagonal indicates discrepancy between top‐1, top‐5, and top‐10 based ranking. Whereas for predictors the behavior is linear, for scorers the data points are much more scattered. Linear regression fits indeed show a strong correlation for predictors, with a correlation coefficient nearing unity, but this value is only 0.32 for the scorer data. These data might hint at participants being more at ease with scoring their own models than those coming from multiple sources, as is the case in the CAPRI Scoring Experiment, but in any case illustrate the necessity of further developing scoring functions that are robust enough and not sensitive to the details of how the models were produced.

**FIGURE 7:**
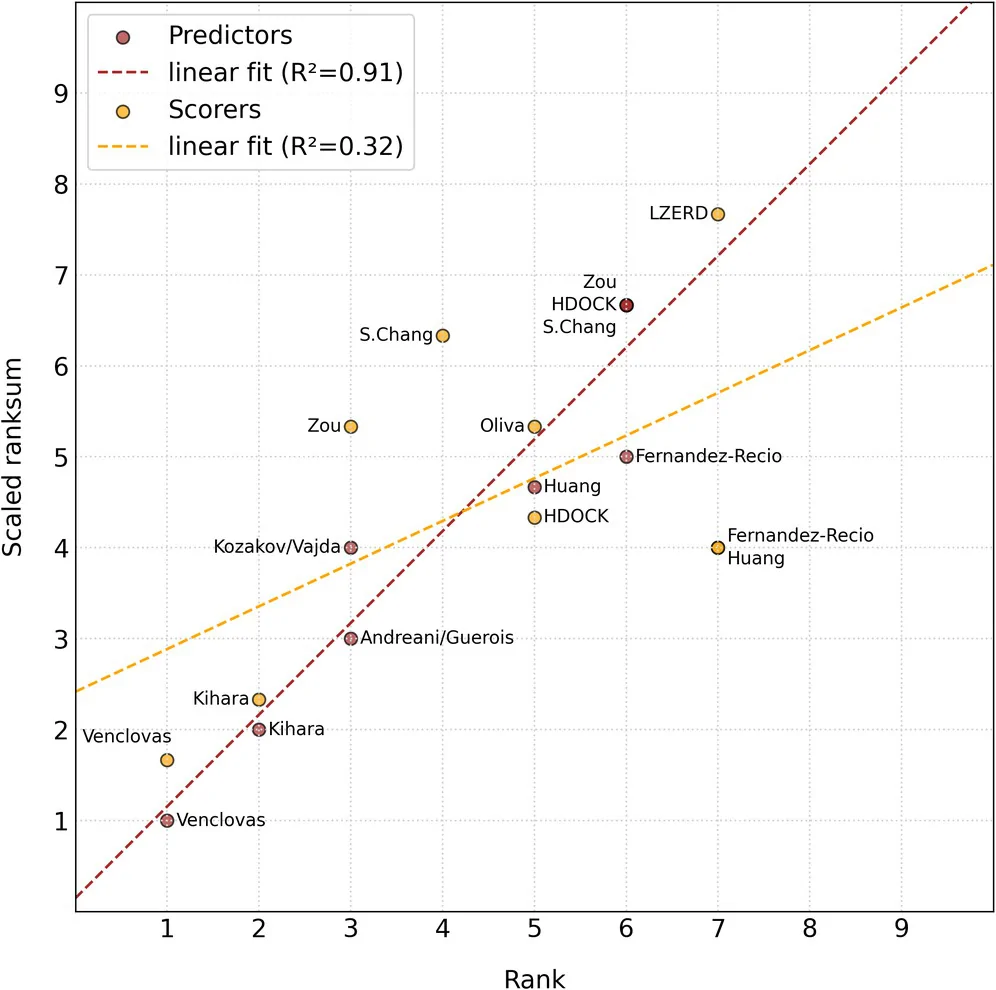
Rank consistency for top predictors and scorers. The x‐axis shows the rank for predictors (brown) and scorers (orange), both based on top‐5 submissions. The y‐axis shows the average rank over the top‐1, top‐5 and top‐10 submissions. Only the first nine predictors and scorers are included. The dashed lines are linear regression fits calculated through the points; their correlation coefficients are shown in the legend.

### 6.4 Performance of Prediction Servers

From the server ranking in Table 4B, we note a poor performance overall on the difficult pre‐AlphaFold targets, with only LZerD and GalaxyPPDock managing an acceptable model for a single subtarget, in both cases thanks to the correct prediction of one of the interfaces of a larger assembly (T163 for LZerD and T160 for GalaxyPPDock). The ranking is therefore dominated by the four post‐AlphaFold targets, three of which included antibodies. HDOCK stands out, having achieved medium quality for three of the four targets, followed by LZerD and ClusPRO, which both managed two medium‐quality submissions. Only a selected few predictor groups achieved 3** on the post‐AlphaFold targets: Venclovas, Zou, and Huang, the last being the author of HDOCK.

## 7 Conclusion and Discussion

The CAPRI 8th Evaluation results were originally presented at the 8th CAPRI assessment meeting, which took place in Grenoble, France, in February 2024, kindly organized by *Sergei Grudinin* and local co‐organizers. The assessment presented here has been carried out over 11 target complexes representing a total of 24 evaluated interfaces. The large difference between the number of targets and evaluated interfaces comes mainly from T160, which was basically 3 targets combined into one (the domain organization and two nanobodies binding to it), but also the domain organization itself was split into six subtargets. All but one target belonged to the difficult prediction category, which is clearly reflected in the absence of any high‐quality model from both predictors and scorers in both the pre‐ and post‐AlphaFold eras (in fact all targets would have been considered difficult if AlphaFold would not have become available). This reflects the challenging nature of the CAPRI endeavor, which, over the years, has been and remains a constant catalyst of new developments in the prediction of biomolecular complexes.

Overall, the CAPRI community is very much alive with about 50 predictor human groups and 10 servers participating and only a slightly lower number for the scoring rounds. We do, however, observe a slight drop in the post‐AlphaFold era. Despite the rise of the AI, the best predictors remain human teams, with *Venclovas* leading in both the predictions and scoring categories, closely followed by the predictor *Kihara* and scorer *Huang* groups. The latter two groups are also the ones developing the top 2 ranked servers HDOCK (Huang) and LZerD (Kihara), which are participating in both prediction and scoring rounds.

The most striking event in this assessment period has been the public and open‐source release of AlphaFold and its model weights. All groups have embraced and integrated in one way or another the new AI tools in their prediction pipelines. While our post‐prediction analysis using AlphaFold indicates a high performance of AlphaFold over the 21 evaluated targets with 14 acceptable or better predictions (about twice the number from best human predictors), this number is biased by a few pre‐AlphaFold targets with multiple interfaces. Focusing on the post‐AlphaFold targets, the situation is different, with the best performance still being obtained by human predictor groups. Furthermore, in this evaluation round, the few antibody and nanobody targets, which typically lack any conservation in their binding regions, clearly reveal the limitations of AlphaFold for these kinds of targets. Future CAPRI rounds will have to show if more recent AI models like, for example, AlphaFold3, do perform better for this type of complexes. Another development which might boost the prediction performance in the future is adaptations of the sampling strategies, as done, for example, in MassiveFold [20] and by various CAPRI predictor groups [34, 35, 36, 37, 38]. This, however, comes at extremely high computational costs and might be difficult to implement in CAPRI settings where targets can be released for prediction at any time during the year and on short notice. Furthermore, with such large datasets of models, scoring might remain the main limiting factor, as indicated in the latest CASP16 assessment meeting, the analysis of which will be published later this year. Also in this CAPRI Evaluation Round scorers were in most cases not able to select the best quality models from the scoring sets.

Next to the CAPRI community, which continues relentlessly to participate in this experiment despite the frustration of tackling such difficult targets, we wish to acknowledge here all the experimentalists providing structures to CAPRI prior to publication. Without those invaluable contributions over many years, the field would not be where it currently stands. We surely hope that the structural biology community at large will continue to provide targets, especially in the areas where the performance of current AI tools is still limited, like obviously the antibody and nanobody complexes, but also other types of complexes involving (flexible) peptides, glycans, and nucleic acids, in particular RNA complexes whose prediction still remains extremely challenging.

## Author Contributions

**Marc F. Lensink:** conceptualization, investigation, writing – original draft, methodology, validation, visualization, writing – review and editing, software, project administration, data curation, resources, supervision. **Nessim Raouraoua:** writing – review and editing, software, methodology. **Guillaume Brysbaert:** writing – review and editing, software, methodology. **Sameer Velankar:** writing – review and editing, resources. **Shoshana J. Wodak:** writing – review and editing, supervision, conceptualization, methodology, project administration, validation, investigation. **Alexandre M. J. J. Bonvin:** writing – review and editing, project administration, conceptualization, methodology, supervision, validation, investigation, writing – original draft.

## Data Availability

The data that support the findings of this study are available from the corresponding author upon reasonable request.
